# Epigenetic Signaling of Cancer Stem Cells During Inflammation

**DOI:** 10.3389/fcell.2021.772211

**Published:** 2021-10-15

**Authors:** Zaoqu Liu, Yuqing Ren, Lingfang Meng, Lifeng Li, Richard Beatson, Jinhai Deng, Tengfei Zhang, Junqi Liu, Xinwei Han

**Affiliations:** ^1^Department of Interventional Radiology, The First Affiliated Hospital of Zhengzhou University, Zhengzhou, China; ^2^Interventional Institute of Zhengzhou University, Zhengzhou, China; ^3^Interventional Treatment and Clinical Research Center of Henan Province, Zhengzhou, China; ^4^Department of Respiratory and Critical Care Medicine, The First Affiliated Hospital of Zhengzhou University, Zhengzhou, China; ^5^Department of Ultrasound, Zhengzhou Sixth People’s Hospital, Henan Infectious Disease Hospital, Zhengzhou, China; ^6^Internet Medical and System Applications of National Engineering Laboratory, Zhengzhou, China; ^7^School of Cancer and Pharmaceutical Sciences, King’s College London, London, United Kingdom; ^8^Richard Dimbleby Laboratory of Cancer Research, School of Cancer and Pharmaceutical Sciences, King’s College London, London, United Kingdom; ^9^Department of Oncology, The First Affiliated Hospital of Zhengzhou University, Zhengzhou, China; ^10^Department of Radiation Oncology, The First Affiliated Hospital of Zhengzhou University, Zhengzhou, China

**Keywords:** cancer stem cells, epigenetics, inflammation, inflammatory factors, signaling pathway

## Abstract

Malignant tumors pose a great challenge to human health, which has led to many studies increasingly elucidating the tumorigenic process. Cancer Stem Cells (CSCs) have profound impacts on tumorigenesis and development of drug resistance. Recently, there has been increased interest in the relationship between inflammation and CSCs but the mechanism underlying this relationship has not been fully elucidated. Inflammatory cytokines produced during chronic inflammation activate signaling pathways that regulate the generation of CSCs through epigenetic mechanisms. In this review, we focus on the effects of inflammation on cancer stem cells, particularly the role of signaling pathways such as NF-κB pathway, STAT3 pathway and Smad pathway involved in regulating epigenetic changes. We hope to provide a novel perspective for improving strategies for tumor treatment.

## Introduction

Cancer remains one of the most devastating diseases in the world. For decades, the occurrence and progression of tumors has been attributed to abnormal genetic changes such as mutations and chromosomal instabilities. However, recent advances in genome sequencing technologies and epigenetic analysis have led to the discovery that epigenetics play critical roles in the regulation of biological characteristics of cells and their malignant transformation (Dawson and Kouzarides, 2012). Furthermore, the identification of cancer stem cells (CSCs) and their association with chemoresistance and tumor relapse has also been a key discovery in the study of cancer (Visvader, 2011). Epigenetic mechanisms have been shown to play a significant role in the development of CSCs. On the other hand, inflammation has also been associated with tumorigenesis. Inflammation is a beneficial response of the immune system to tissue damage and pathogens. However, prolonged immune response leads to chronic inflammation that can promote malignant transformation of cells (Elinav et al., 2013). Several studies have demonstrated that chronic inflammation is involved in tumor development and progression. This has led to the emergence of a new field of cancer research involving the regulation of CSCs by chronic inflammation. Chronic inflammation can regulate the proliferation, metabolism, and differentiation of tumor cells as well as the self-renewal ability of CSCs by inducing secretion of inflammatory factors, oxidative stress and hypoxia. Therefore, it is crucial to understand the interaction between inflammatory factors and CSCs. Recently, epigenetic mechanisms have been shown to regulate inflammation and the generation of CSCs in cancer, and several molecular mechanisms underlying these processes have been elucidated. Importantly, epigenetic mechanisms have been associated with the variability observed in therapeutic responses. Therefore, an in-depth analysis of the relationship among CSCs, epigenetics and inflammation is not only critical in the understanding of tumor characteristics, but it is also key in the development of therapeutic strategies against tumors. In this review, we focus on the characteristics of CSCs, epigenetic clues, and the effects of epigenetic signaling pathways, particularly the effects of inflammation related factors on tumorigenesis, progression, and therapeutic response. We also discuss recent advances in targeting CSCs using epigenetic therapies.

## Cancer Stem Cells

Cancer stem cells are small subsets of cells with capacity for self-renewal and the ability to differentiate into the different cell types that constitute a tumor (Reya et al., 2001). Therefore, these cells have the same stem cell-like properties as normal tissue stem cells. CSCs also exhibit capacity for spheroid formation, migration, invasion, and development of drug resistance, thus contributing to tumor cell proliferation. It should be noted that a general differentiation capacity is not an obligatory feature of CSCs. The ability of CSCs to differentiate and repopulate cell types found in the original tumor is of greater significance.

In 1937, Furth and Kahn successfully engrafted single mouse leukemia cells that developed into leukemia (Furth et al., 1937), demonstrating for the first time the existence of cancer cells with stem cell-like properties. These cancer cells are now termed cancer stem cells or tumor-initiating cells. In the early 1990s, human leukemic stem cells were identified and transplanted by Dr. John Dick (Furth et al., 1937; Lapidot et al., 1994). Similarly, recent studies have shown that CSCs also play a critical role in the development of several solid tumors, including prostate, brain, colon, pancreatic, and breast cancers (Chen et al., 2012; Meacham and Morrison, 2013). This has led to the emergence of a field of research in cancer stem cells which has increased the understanding of cancer and renewed the hope of cancer eradication. CSCs arise due to mutations in normal stem cells, cancer cells that have undergone epithelial-mesenchymal transition (EMT), or dedifferentiated somatic cells. However, the molecular mechanisms underlying the development of CSCs have not yet been well elucidated. Recent reports have revealed the role of microRNAs (miRNAs) in regulating CSCs (Liu et al., 2011; Asadzadeh et al., 2019; Khan et al., 2019). In addition, several cell surface markers, including CD24, CD44, CD133 and aldehyde dehydrogenase 1 (ALDH1), have been identified, classified and used to isolate CSCs (Yu et al., 2012). Evidence shows that these markers, especially those on the surface of CSCs, are cancer specific (Hao et al., 2014). Moreover, cancer stem cells are one of the major causes of tumor heterogeneity and acquired drug resistance.

Overall, CSCs have the potential for self-renewal and heterogeneous differentiation, leading to tumor formation (Krishnamurthy and Nor, 2012). CSCs can also arise when epigenetic reprogramming induces dedifferentiation of normal cells resulting in the cells acquiring stem cell-like properties. A major difference between normal stem cells and cancer stem cells is their ability to modulate stemness pathways. In normal stem cells, stemness pathways including TGF-β, Wnt/β-Catenin, notch, and Janus kinase/signal transducer and activator of transcription, among others, are tightly controlled by complete genetics or epigenetics. In contrast, the regulation of these pathways in CSCs is not tightly controlled, and their deregulation as well as inappropriate interactions among the pathways may contribute to the propagation and pathogenicity of CSCs. In addition, cells in the tumor microenvironment, such as cancer-associated fibroblasts, can trigger growth and differentiation of CSCs by secreting growth factors or activating signaling pathways through cell-cell interactions (Lopez-Lazaro, 2015; Batlle and Clevers, 2017; Asadzadeh et al., 2019).

## Inflammatory Microenvironment and Cancer Stem Cells

**FIGURE 1:**
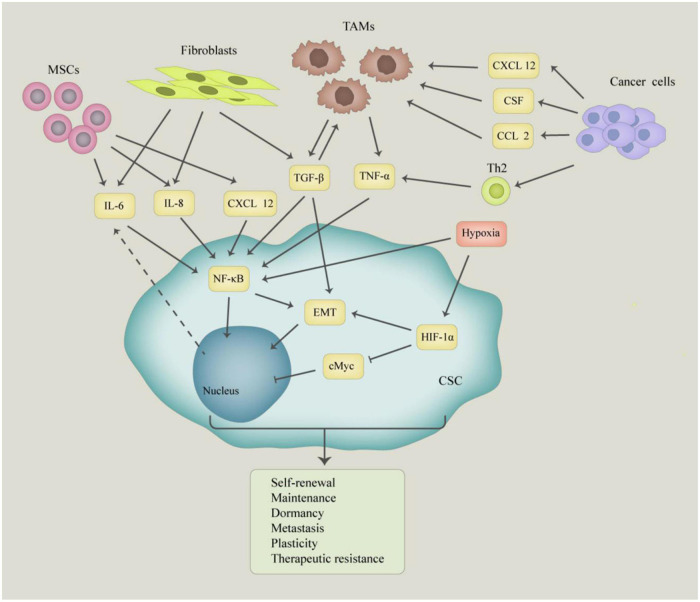
The molecular and cellular basis of interaction between cancer stem cells (CSCs) and tumor microenvironment. The CSCs interact with mesenchymal stem cells (MSCs), tumor-associated macrophages (TAMs), fibroblasts, and cancer cells, which produce a large number of growth factors and cytokines, such as IL-6, IL-8, TGF-β, and TNF-α. Those factors play an important role in inducing self-renewal, plasticity, dormancy, metastasis, and tumorigenesis of CSCs.

Inflammation is a self-limiting physiological response that mediates the repair of damaged tissues. However, chronic inflammation has been associated with several human diseases, including cancer (Hu et al., 2017). The microenvironment of normal stem cells as well as cancer stem cells is critical for their maintenance and function. Inflammatory factors present in the tumor microenvironment control tumor initiation, progression, and the nature of CSCs (Plaks et al., 2015; Figure 1). Inflammation promotes the acquisition and maintenance of the cancer stem cell phenotype by stimulating EMT (Rhim et al., 2012). The secretion of inflammatory cytokines creates an inflammatory microenvironment which also leads to the generation of CSCs (Scheel and Weinberg, 2012; Nieto, 2013; Nieto et al., 2016). Current studies show that the maintenance of the cancer stem cell phenotype and function is more complex than previously thought. Therefore, it is important to elucidate the signaling pathways that regulate cancer stem cell phenotype and function. During their growth, tumors recruit various types of immune cells into the tumor microenvironment. These immune cells change their phenotype and function within the tumor cells to promote tumor growth and metastasis. Additionally, these infiltrating immune cells cause inflammation in the tumor tissue by inducing the secretion of growth factors, cytokines, chemokines, proteases, and extracellular matrix modifying enzymes (Bierie and Moses, 2010). For instance, transforming growth factor-β induces the EMT phenotype in breast cancer which confers stem cell-like properties to normal and transformed mammary epithelial cells (Hollier et al., 2009). These inflammatory cells, together with the active factors they secrete, shape the microenvironment on which CSCs depend on for their generation and their ability to stimulate and protect cancer stem cells to maintain their self-renewal ability.

Tumor-associated macrophages (TAMs) play a crucial role in the development of cancer (Biswas et al., 2013). TAMs are the main types of infiltrating immune cells in breast cancer as well as malignant glioma tissues, accounting for approximately 5 to 50% of all cells in the tumor tissues. These TAMs secrete a number of factors, including interleukin-1 (IL-1) and tumor necrosis factor-α (TNF-α), which have been shown to promote tumor invasion and metastasis (Biswas et al., 2013). TAMs also secrete other cytokines such as IL-6, IL-8, and CSF2, which maintain the stemness of tumors through paracrine mechanisms (Lu et al., 2014). In addition to various factors secreted by immune cells and stromal cells in the tumor microenvironment to regulate cancer stem cells, cytokines secreted by tumor cells can also act on cancer stem cells in an autocrine manner. Recently, Iliopoulos et al. (2011) reported that the pro-inflammatory cytokine, IL-6, can transform non-cancer stem cells into cancer stem cells. Colorectal cancer cells exposed to IL-6 suppressed EMT transcription factor expression, as well as the invasion, and metastasis of the cancer cells by repressing IL-6 through expression of miR-34a (Rokavec et al., 2014). In transformed breast cells, IL-6 reduces the expression levels of let-7 by activating Lin28 transcription *via* the NF-κB pathway, leading to the development of breast cancer and enhanced CSC characteristics (Iliopoulos et al., 2009; Shyh-Chang and Daley, 2013). IL-6 also promotes the expression of miR-203 in stem cells by downregulating the EMT activator ZEB1 thus linking EMT activation to stemness maintenance (Wellner et al., 2009). In addition, HER2 + breast cancer cells that develop resistance to trastuzumab treatment are enriched with CSCs, exhibit EMT features and express high levels of IL-6. Blockade of the IL-6 receptor reduced the tumor proliferative capacity of these cells, further indicating that IL-6 regulates CSCs through an autocrine loop (Korkaya et al., 2012). Long et al. (2012) reported that ovarian cancer stem cells promote tumor metastasis by expressing high levels of CCL5, activating the NF-κB signaling pathway in an autocrine manner and up-regulating the expression of MMP-9. Ginestier et al. (2010) reported that breast cancer stem cells highly expressed CXCR1 receptor, and that IL-8 (a ligand of CXCR1) was able to enhance the proportion of CSCs and maintain the stemness of CSCs. The communication between tumor cells and the tumor environment is bidirectional. The factors secreted by cancer stem cells attract necessary cells to their environment, and these cells secrete factors that are beneficial to cancer stem cells, thereby coexisting harmoniously.

## Effects of Inflammatory Factors on Epigenetic of Cancer Stem Cells

Epigenetic regulation of the genome is one of the main mechanisms of altering the genetic code to control the hierarchy of cell development. Epigenetic mechanisms refer to changes in gene expression that are not caused by changes in DNA sequence but by changes in DNA methylation, histone modifications, chromatin remodeling, and non-coding RNA, such as microRNAs (miRNAs). These epigenetic mechanisms control the genetic landscape and dictate cell fate (Hu et al., 2017). Such changes in the genome are very important during normal mammalian development and differentiation of ESCs (Reik, 2007), and any alteration in epigenetic signaling can affect the accumulation of cells with stemness and self-renewing capacity, to produce CSCs. DNA hypermethylation of cytosines in CpG dinucleotides in various cancers has been associated with silencing of tumor suppressors and genes regulating differentiation (Esteller, 2007). A decrease in the expression of these two types of genes may contribute to the development of CSCs in the tumor cell populations (Jones and Baylin, 2007; Ohm et al., 2007). DNA methylation silences transcription by recruiting methyl-CpG binding domain proteins that are able to induce histone modifying enzymes to promote repressive histone modifications (Wade et al., 1999; Baylin et al., 2011; Easwaran et al., 2014), or to a lesser extent, facilitate expression by preventing access to transcription factors (Bell and Felsenfeld, 2000; Hark et al., 2000). DNA methylation is regulated by three DNA methyltransferases (DNMT1, DNMT3a, and DNMT3b) (Bird, 2002). Actually, it has been reported that DNA methylation plays an important role in maintaining the characteristics of CSCs in leukemia, lung, and colon stem cells (Broske et al., 2009; Morita et al., 2013; Liu C.C. et al., 2015). Moreover, inflammation can promote tumorigenesis by inducing epigenetic alterations in cells. In most cases, epigenetic inheritance cannot be explained by a single alteration, but by the interaction of different epigenetic mechanisms. Many inflammatory factors regulate the DNA methylation patterns that induce cancer initiation and progression in cancers such as gastric cancer, ovarian cancer, and liver cancer. IL-1β is a proinflammatory cytokine that enhances the activity of DNMT through the production of nitric oxide in gastric cancer, leading to CpG methylation-mediated gene silencing (Hmadcha et al., 1999). Additionally, TGF-β can induce the expression and activity of DNMTs, leading to integral changes in DNA methylation during the EMT of ovarian cancer (Cardenas et al., 2014) and the acquisition of stemness by cancer cells. In breast cancer, TGF-β induces endothelial cell transformation by recruiting DNMT and histone methyltransferases (EHMT2 and SUV39H1) to the CDH1 gene promoter, which leads to the development of CSCs (Dong et al., 2012, 2013; David and Massague, 2018). Epigenetic processes also control the IL-6-mediated induction of cancer cell stemness (Drost and Agami, 2009; Iliopoulos et al., 2009, 2010a). It has been shown that p53 recruits DNMT1 to the promoter of its target genes (Esteve et al., 2005). The deletion of p53 leads to the demethylation of the IL-6 promoter thus activating IL-6 signaling. Subsequently, IL-6 signaling upregulates DNMT1 which methylates the promoter of the p53 gene (Hodge et al., 2005, 2007; Liu C.C. et al., 2015), thus initiating an IL-6 autocrine loop (D’Anello et al., 2010). Therefore, the activation of this IL-6 autocrine loop drives cancer cells toward a stem-like phenotype through epigenetic reprogramming (D’Anello et al., 2010; Liu C.C. et al., 2015). IL-6 also facilitates hypermethylation of the promoter of the miR142-3p gene, thereby inhibiting the expression of miR-142-3p. These effects promote cell stemness and invasiveness in glioblastoma (Chiou et al., 2013). Thus, cytokine signaling promotes CSCs through epigenetic mechanisms. More importantly, inflammatory cytokines such as TGF-β, TNF-α, IL-1, and IL-6 activate signal transduction pathways such as Smads, NF-κB and STAT3 pathways by recruiting epigenetic regulators. These inflammatory pathways are interconnected and produce molecular regulatory circuits and networks to control the generation and maintenance of CSCs.

## Nuclear Factor-κB Pathway

**FIGURE 2:**
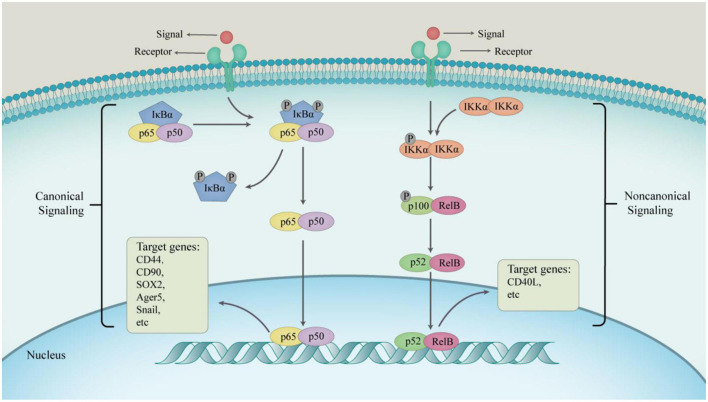
The progress of two main NF-κB signaling pathways. In a canonical way, the main physiological function of NF-κB is the p50–p65 dimer. The active p50–p65 dimer is further facilitated by post-translational modification and accumulates in the nucleus to regulate the expression of target genes in combination with other transcription factors. In non-canonical way, IKKα homodimers transform RelB–p100 dimer into RelB–p52 dimer driven by signaling factors, which activate non-canonical NF-κB target genes to express consequently.

Nuclear factor-κB (NF-κB) is an inducible transcription factor that regulates the transcription of its target genes (Zhang et al., 2017), and plays key roles in inflammatory responses and cancer development (Hoesel and Schmid, 2013). The NF-κB family of transcription factors regulate inflammation, self-renewal or maintenance and metastasis of immune cells and CSCs. Furthermore, the NF-κB pathway is also involved in cell survival, proliferation, and differentiation (Hayden and Ghosh, 2008), and is considered to play key roles in the many steps involved in cancer initiation and progression. Moreover, cytokines, growth factors, angiogenic factors, and proteases produced during tumor development and progression can activate the NF-κB signaling pathway (Figure 2). The NF-κB family consists of five members: NF-κB1, NF-κB2, c-Rel, RelB, and p65. The members of this family have a conserved N-terminal Rel homologous domain that is involved in nuclear localization, DNA binding, homodimerization, and heterodimerization. The p50-p65 dimer is the primary functional form of NF-κB (May and Ghosh, 1997; Hayden and Ghosh, 2008; Hoesel and Schmid, 2013), which is mainly regulated through subcellular localization. The activation of the NF-κB pathway involves the translocation of the transcription factor complex from the cytoplasm to the nucleus (Novack, 2011). The activity of the transcription factor multiplex is regulated through either canonical NF-κB signaling or the non-canonical NF-κB signaling. The canonical NF-κB signaling pathway is activated by ligands (such as bacterial components, IL-1β, TNF-α, and lipopolysaccharide) binding to their respective receptors (such as toll-like receptors, IL-1 receptors, TNF receptors, and antigen receptors) (Perkins and Gilmore, 2006). Activation of these receptors leads to the phosphorylation and activation of IκB kinase (IKK), which subsequently initiates phosphorylation of IκB proteins. The receptors involved in the activation of the non-canonical pathway include receptor activator for NF-κB, B cell activating factor, CD40, and lymphotoxin β receptors (Sun, 2011). This pathway activates NF-κB through the inducible kinase (NIK), which predominantly phosphorylates and activates IKK1. IKK1 then induces the phosphorylation of p100 to generate p52 (Xiao et al., 2001).

In esophageal cancer, the up-regulation of PLCE1 oncoprotein through epigenetic mechanisms drives esophageal cancer angiogenesis and proliferation by activating the NF-κB signaling pathway. Hypomethylation-induced PLCE1 in esophageal squamous cell carcinoma (ESCC) cohorts can bind and phosphorylate p65 and IκBα proteins. Subsequently, phosphorylated IκBα promotes nuclear translocation of p50/p65 and p65, where they act as transcription factors for vascular endothelial growth factor-C and bcl-2, driving tumor angiogenesis and inhibits apoptosis *in vitro* (Chen et al., 2019). Likewise, Sox9 is demethylated in pancreatic cancer stem cells and is involved in the invasion process. Several studies have identified a potential NF-κB binding site on the Sox9 promoter, and demonstrated that the p65 subunit of NF-κB positively regulates Sox9 expression by directly binding to its promoter (Sun et al., 2013). Inhibition of DNA of methyltransferase activity causes demethylation of the Sox9 promoter, which leads to the enrichment of p65 on the Sox9 promoter and up-regulation of Sox9 expression. These results indicate that regulation of Sox9 by NF-κB is dependent on its methylation status, and demethylation may enhance NF-κB binding to the Sox9 promoter (Sun et al., 2013). In skin cancer, Ras/NF-κB-induced epigenetic silencing of Let-7c, an upstream regulator of NF-κB, causes HaCaT cells to acquire cancer stem cell-like properties and undergo neoplastic transformation (Jiang R. et al., 2014). In addition, MiRNAs have recently been reported to regulate the NF-κB signaling pathway. For instance, MiR-210-3p maintains sustained activation of NF-κB signaling by targeting TNIP1 and SOCS1, negative regulators of NF-κB signaling, leading to EMT, invasion, migration, and bone metastasis of prostate cancer cell (Ren et al., 2017b). MiR-372/373 enhances the stemness features of colorectal cancer by acting on various signaling mechanisms associated with stem cell differentiation. It can inhibit the NF-κB, MAPK/Erk, and VDR signaling pathways that are essential for differentiation (Khan et al., 2019). Downregulation of miR-136 stimulates CSCs by activating various proteins including NF-κB, survivin, BCL2, CyclinD1, and BCL2. On the other hand, upregulation of miR-155 activates NF-κB (Khan et al., 2019).

## STAT3 Pathway

The JAK/STAT3 signaling pathway plays crucial roles in various types of cancer. Activation of this pathway enhances EMT leading to increased tumorigenic and metastatic capacity, development, transition of CSCs, and chemoresistance of cancer. The STAT family has seven members (STAT1, 2, 3, 4, 5α, 5β, and 6). Each STAT protein has highly conserved amino terminal, SH2, coiled-coil, DNA binding, and transactivation domains (Darnell, 1997). The SH2 domain is indispensable for the interaction between STAT proteins and STAT cytokine receptors. This is because the cytokine receptors recognize and bind to tyrosine residues on the SH2 domain. The SH2 domain is also essential in the formation of stable homodimers or heterodimers with other STAT proteins (Heim et al., 1995; Hemmann et al., 1996). Cytokines induce dimerization of STAT3 *via* acetylation of Lys685 in the SH2 domain of STAT3, which is mediated by histone acetyltransferase p300 (Yuan et al., 2005). Other transcription factors interact with the Asp170 residue in the coiled-coil domain of STAT3 (Zhang et al., 1999), and their receptor binding, DNA binding, nuclear translocation and dimerization all require IL-6-induced tyrosine phosphorylation of STAT3 (Zhang et al., 2000). In addition, the N-terminal domain of STAT3 has various functions including stabilization of STAT3 tetramers, nuclear translocation, cooperative DNA binding, and protein-protein interactions (Hu et al., 2015).

STAT3 also induces the transcription of genes involved in the inflammatory response. Sakamoto et al. (2016) found that Janus kinase 1 (Jak1) played a significant part in inflammatory cytokine signaling and remodeling of the mammary gland. STATs are also involved in the regulation of apoptosis, differentiation, and stem cell maintenance. STAT3 and STAT5 have been shown to play different roles in breast cancer. Walker et al. (2014) reported that STAT5 regulates STAT3 and they both play a role in breast cancer progression. Intestinal inflammation also promotes tumorigenesis by enhancing tumor cell survival and proliferation. Members of the IL6, IL10/IL22, and IL17/IL23 cytokine families have been implicated in this process. They all bind to their receptors, leading to persistent, non-transient activation of STAT3, which not only promotes the growth of malignant cells but also inhibits the anti-tumor effects of both innate and acquired immune cells. This allows inflammation-associated and sporadic gastrointestinal tumors to grow (Ernst et al., 2014). STAT3 also controls the fate of prostate cancer cells, the interaction of tumor cells with the microenvironment, and maintains the number of CSCs (Kroon et al., 2013). JAK2/STAT3 signaling also plays an important role in promoting colorectal stem cell persistence and radio-resistance by inhibiting apoptosis and enhancing clonogenic potential (Park et al., 2019). Upregulation of IL-6 in colorectal cancer-derived mesenchymal stem cells (CC-MSCs) leads to enhanced metastasis and survival of colorectal cancer patients by activating PI3K/Akt *via* the IL-6/JAK2/STAT3 signaling pathway (Ma et al., 2019).

STATs not only act as transcriptional inducers, but they also induce epithelial mesenchymal transition and generate an oncogenic microenvironment (Li et al., 2011). Dr. Lee reported that non-canonical signaling by histone-lysine N-methyltransferase EZH2 in regulating STAT3 methylation is required for glioblastoma stem cell maintenance (Kim et al., 2013). IL-6 through STAT mediated induction of cancer cell stemness is also controlled by epigenetic processes (Drost and Agami, 2009; Iliopoulos et al., 2009, 2010a,2010b; Zahnow and Baylin, 2010; Rokavec et al., 2016). The tumor suppressor gene p53 recruits the DNMT1 methylase to the promoters of its target genes (Esteve et al., 2005). The loss of p53 expression is associated with the demethylation of the IL-6 promoter, which initiates an IL-6 autocrine loop (D’Anello et al., 2010). IL-6 signaling upregulates DNMT1 which in turn methylates the promoter of the p53 gene (Hodge et al., 2005, 2007; Liu C.C. et al., 2015). It also leads to the activation and acetylation at Lys685 of STAT3 in tumor cells, which is critical for the inactivation of tumor suppressor genes through the methylation of their promoters (Lee et al., 2012). Thus, the generation of this autocrine IL-6 loop induces epigenetic reprogramming that drives cancer cells to acquire a stem cell-like phenotype (D’Anello et al., 2010; Liu C.C. et al., 2015). Additionally, miRNAs can stimulate or inhibit the function of CSCs. IL-6/STAT3 mediated miR-200c transformation and inhibited EMT process in breast (Rokavec et al., 2012) and lung (Zhao et al., 2013) epithelial cancer cells. More importantly, the up regulation of miR-196b-5p and miR-500a-3p as well as the down-regulation of miR-218 activates STAT3 molecule. MiR-196b-5p plays a central role in maintaining CSCs characteristics associated with resistance to cancer therapy by targeting STAT3 signaling pathway in colorectal cancer stem cells (Ren et al., 2017a). Downregulation of miR-218 in lung cancer cells can induce constitutive activation of STAT3, which is closely associated with tumorigenesis. In ALDH positive lung CSCs, aberrant expression of miR-218 upregulated IL-6/JAK-STAT3 signaling and stemness features of tumors (Yang et al., 2017). Similarly, miRNAs have been associated with aberrant activation of the STAT pathway in breast and colorectal cancers (Ren et al., 2017a; Vahidian et al., 2019).

## Smad Pathway

Smad transcription factor mediates signal transduction by the TGF-β cytokine superfamily to control a variety of cell responses, including development, stem cell maturation and carcinogenesis (Massague, 2012). It also plays a variety of roles in embryonic development, adult tissue regeneration and homeostasis, such as cell proliferation, differentiation, apoptosis and dynamic balance. There are at least nine Smad proteins that are divided into three subfamilies based on their structure and function: receptor activated or pathway restricted Smads (R-Smads), common Smads (Co-Smads), and inhibitory Smads (I-Smads) (Moustakas et al., 2001; Brown et al., 2017). Smad proteins regulate the transcription of their target genes by binding to specific DNA sequences in the promoter regions and recruiting either co-activators or co-repressors (Massague et al., 2005). Smad3 is the major effector of TGF-β-mediated endothelial cell transformation or differentiation. Ligand binding to the TGF-β receptor complex on the cell surface induces phosphorylation of the C-terminal of type I receptor and activates Smad2 and Smad3. These two the then form an isomer complex with Smad4, translocate into the nucleus, and interact with bound transcription factors to activate or inhibit TGF-β/Smad target genes (Singh et al., 2019). Co-Smads such as Smad4 mediate TGF-β signal transduction processes, while I-Smads, such as Smad6 and Smad7, regulate signaling by the TGF-β family by binding to activated type I receptors (Massague et al., 2005).

TGF-β1/Smad pathway is closely associated with tissue fibrosis (Xu et al., 2016; Hu et al., 2018; Ma and Meng, 2019). Several studies have reported that TGF/Smad signaling is activated in human cancers. The pathway also plays an important role in the proliferation of CSCs. In breast cancer and cutaneous squamous cell carcinoma, TGF-β has been shown to initiate tumor formation and promote the generation of CSCs (Pang et al., 2018; Najafi et al., 2019; Singh et al., 2019). Moreover, recent studies have reported that TGF-β1 is associated with the malignant behavior of tumors (Pang et al., 2018; Najafi et al., 2019). For example, high expression of TGF-β1 regulates EMT-related genes through Smad signaling, thereby promoting the progression of colorectal cancer. Recent research studies have suggested that epigenetic mechanisms play a critical role in the TGF-β1/Smad pathway. Tang et al. (2015) found that profilin-2 (Pfn2) enhances Smad2 and Smad3 expression through epigenetic mechanisms in lung cancer. Profilin-2 inhibits the recruitment of histone deacetylase (HDAC) to the Smad2 and Smad3 promoters by preventing the nuclear translocation of HDAC1 at the C-terminus of these proteins. This leads to the transcriptional activation of Smad2 and Smad3, which increases their expression levels and promotes lung cancer growth and metastasis (Tang et al., 2015). TGF-β induces epigenetic regulation of the hepatoma stem cell marker CD133 through the Smad pathway. TGF-β1 also regulates the expression of CD133 by inhibiting the expression of DNMT1 and DNMT3b, which in turn leads to the demethylation of the CD133 promoter. HCC cells containing CD133 with demethylated promoters are characterized by chemoresistance, self-renewal, and multilineage differentiation (You et al., 2010). Similarly, HDAC dependent epigenetic modifications regulate the TGF-β/Smad pathway in glioblastoma (GBM), which plays an important role in GBM tumorigenesis, resistance to common therapies and poor clinical outcomes (Sferra et al., 2017). In addition to alterations in DNA and histones, miRNAs have also been found to act as epigenetic modulators of the TGF-β/Smad signaling pathway. In CD44 (+) GCSCs, miR-106b regulates TGF-β/Smad signaling to enhance stemness characteristics of GCSCs, including EMT, self-renewal and invasion (Yu et al., 2014). MiR-4666-3p and miR-329 inhibit the stemness of colorectal cancer cells by targeting TGF-β/Smad pathway (Ye et al., 2019). MiR-148a can repress TGF-β/Smad2 signaling pathway in liver cancer stem cells (Jiang F. et al., 2014). Moreover, Smad7 is a newly identified target gene of miR-106b, which acts as an inhibitor of TGF-β/Smad signaling pathway, and suppresses gastric cancer stem cell spheroid formation (Yu et al., 2014).

## Cancer Stem Cells Targeting Therapeutic Using Epigenetic Modifying Drugs

The ability of CSCs to resist therapy is widespread and in the main cause of multidrug resistance in tumors. This ability stems from the increased expression of detoxifying enzymes, and improved activation of survival signaling pathways, DNA repair mechanisms, and drug efflux pumps in CSCs (Dawood et al., 2014; Dzobo et al., 2016). Recently, CSCs have also been shown to undergo epigenetic reprogramming, which makes it difficult to eradicate them in cancer (Dymock, 2016). The involvement of epigenetic mechanisms in CSC formation and maintenance makes epigenetics a potential therapeutic target for CSCs. Therefore, small molecule compound inhibitors with the ability of inducing the differentiation of CSCs are the most promising agents against such tumor cells. Many inhibitors of epigenetic regulatory enzymes such as histone deacetylases (HDACs), histone acetyltransferases (HATS) and DNA methyltransferases (DNMTs), have been extensively studied and are currently in clinical trials for the treatment of several cancers. Furthermore, deregulation of chromatin remodeling has been implicated in tumor initiation and progression, which makes chromatin remodeling proteins effective targets for small molecule inhibitors. Indeed, a large number of these therapeutic strategies intend to induce the differentiation of CSCs and to improve the sensitivity of these cells to chemotherapy, with the ultimate goal of decreasing tumor recurrence and increasing patient survival. Inhibitors of DNA methylation were the first epigenetic drugs tested for cancer treatment (Sharma et al., 2010). The most widely studied DNMT inhibitors include azacitidine and decitabine, which are analogs of cytosine. These molecules are incorporated into DNA and covalently bind to DNA methyltransferases thus inhibiting their functions (Juttermann et al., 1994; Stresemann and Lyko, 2008), and leading to their degradation (Ghoshal et al., 2005). Liu C.C. et al. (2015) showed that inhibition of DNMT1 reduced the proliferation and tumorigenic capacity of lung cancer stem cells. The acetylation of histone tails is mediated by HATS and HDACs. Voinostat and romidessin are HDAC inhibitors that have been approved for the treatment of cutaneous T-cell lymphoma (Olsen et al., 2007; Piekarz et al., 2009). Travaglini et al. (2009) found that the HDAC inhibitor, valproic acid, was able to epigenetically reprogram breast cancer cells to a more “physiological” phenotype, thereby increasing sensitivity to other forms of breast cancer therapy. In the same way, there has recently been increased interest in the research on the epigenetic regulation of CSCs by histone lysine methyltransferase (HKMT) inhibitors and histone demethylase (HDM) inhibitors (Schenk et al., 2012; Liu K. et al., 2015; McGrath and Trojer, 2015). Further, since signaling pathways play crucial roles in promoting the propagation of CSCs, maintaining the CSC phenotype, and embryonic development (Matsui, 2016; Krishnamurthy and Kurzrock, 2018; Pan et al., 2018), therapeutic targets against these pathways have been developed. These signaling pathways include NF-κB, JAK-STAT and TGF/Smad. Specifically, targeting epigenetic changes in signaling pathways has emerged as a new research direction in tumor therapy. For example, tocilizumab inhibits IL-6/STAT3 signaling and suppresses cancer/inflammation epigenetic IL-6/STAT3/NF-κB positive feedback loop, which has tremendous therapeutic value for patients with refractory triple negative breast cancer (TNBC) (Alraouji et al., 2020). Additionally in pancreatic cancer stem cells, activation of the NF-κB pathway relies on methylation of the downstream regulatory gene Sox9, and DNMT inhibitors could perhaps be a new therapeutic strategy for pancreatic cancer treatment (Sun et al., 2013).

## Conclusion

Epigenetic mechanisms play a significant role in the development of cancer stem cells. Likewise, chronic inflammation is closely associated with the initiation and progression of CSCs. These interactive processes influence and promote each other, thereby modulating the self-renewal capacity, drug-resistant properties, and metastatic potential of CSCs. We have discussed several crucial aspects and examples of signaling pathways associated with inflammation and epigenetics which drive or promote tumorigenesis and metastasis, particularly in CSCs. Some therapeutic directions and drugs targeting epigenetic mechanisms are also exemplified in this article. The regulation of epigenetic mechanisms by inflammation plays a key role in CSCs generation. Therefore, a systematic understanding of the signaling pathways associated with epigenetic regulatory mechanisms in the tumor inflammatory microenvironment can give more insights into the process of tumorigenesis. This will help identify novel strategies for tumor therapy.

## Author Contributions

ZL designed the work. YR collected the materials and wrote the manuscript. ZL, XH, TZ, RB, JL, LM, JD, and LL edited and revised the manuscript. All authors have read and agreed to the published version of the manuscript.

## Conflict of Interest

The authors declare that the research was conducted in the absence of any commercial or financial relationships that could be construed as a potential conflict of interest.

## Publisher’s Note

All claims expressed in this article are solely those of the authors and do not necessarily represent those of their affiliated organizations, or those of the publisher, the editors and the reviewers. Any product that may be evaluated in this article, or claim that may be made by its manufacturer, is not guaranteed or endorsed by the publisher.
